# Supplementary material for: Evidence of ferroelectric features in low-density supercooled water from ab initio deep neural-network simulations
Source: Proc Natl Acad Sci U S A. 2024 Jul 31;121(32):e2407295121. doi: 10.1073/pnas.2407295121 (PMC11317578; doi:10.1073/pnas.2407295121)
Supplement: Supplementary file 1 — Appendix 01 (PDF) [file pnas.2407295121.sapp.pdf]

PNAS

Supporting Information for

Evidence of ferroelectric features in low-density supercooled water from *ab initio* deep neural-network simulations

Cesare Malosso,Natalia Manko, Maria Grazia Izzo, Stefano Baroni, Ali Hassanali

Ali Hassanali.
E-mail: ahassana@ictp.it

This PDF file includes:

Supporting text
Figs. S1 to S4
SI References

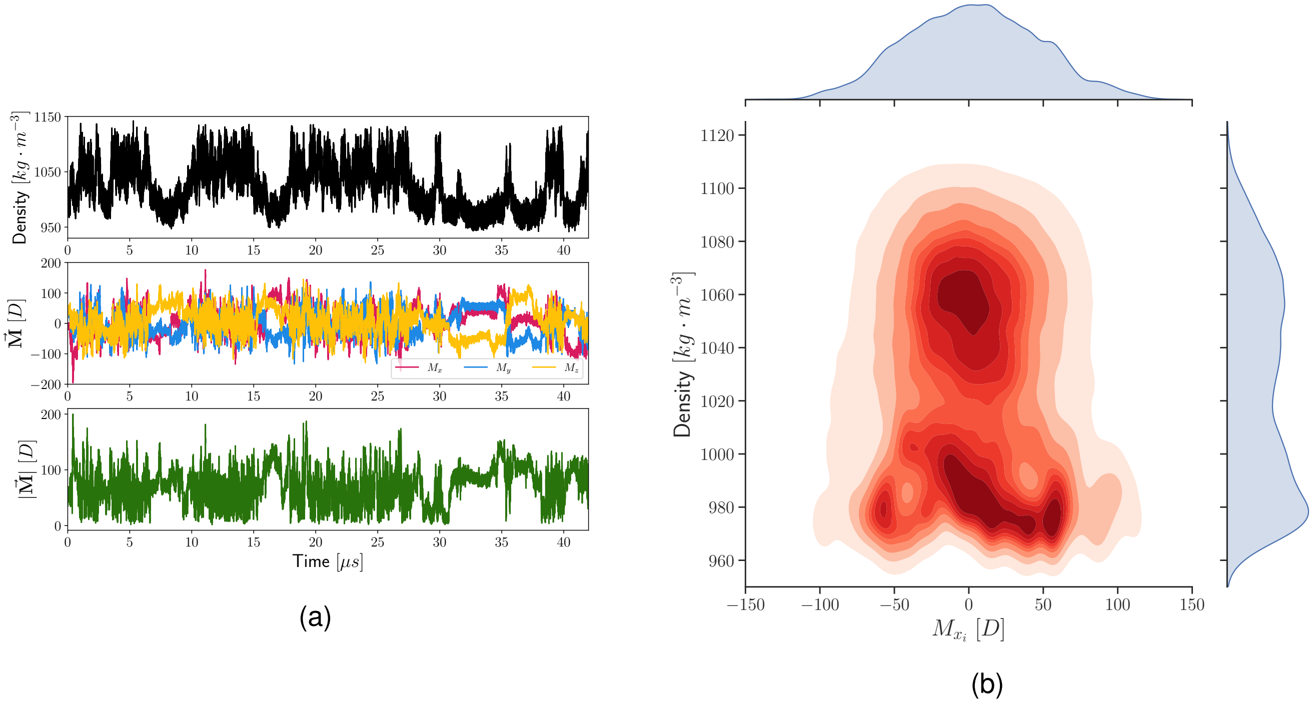

Fig. S1. *(a)*: Density[*top*], total dipole[*middle*] and its modulus[*bottom*] fluctuations during isobaric-isothermal molecular dynamics simulations of TIP4P/2005 at nearly supercritical conditions - 177K and 1750bar. *(b):* Statistics of critical fluctuations displayed as two-dimensional density and dipole probability densities.

Supporting Information Text

1. Results for TIP4P/2005 simulations

We analyzed the coupling between the density and dipole over a microsecond-long TIP4P/2005 water simulation made of 300 molecules at nearly supercritical conditions - 177K and 1750bar (1). The dipole has been computed from the atomic coordinates and the point charges built into the model. The result of this analysis is shown in Fig. S1a. The different subplots show the temporal evolution of the density of the system (*top*), the time series of each component of the total dipole (*middle*), and finally of its modulus (*bottom*). In Figure S1b, the density and the three equivalent components of the dipole are illustrated through a two-dimensional probability distribution.

In Fig. S2 we report the total dipole auto-correlation function (ACF) for both the TIP4P/2005 and the SCAN-DFT simulations (2). The ACF is computed as:

$$ACF(t) = \frac{\langle (\vec{\mathbf{M}}(t) - \langle \vec{\mathbf{M}} \rangle)(\vec{\mathbf{M}}(0) - \langle \vec{\mathbf{M}} \rangle) \rangle}{\langle \vec{\mathbf{M}}^2 \rangle - \langle \vec{\mathbf{M}} \rangle^2}$$

For the HDL phase, the relaxation times associated with the dipole for SCAN and TIP4P/2005 model are 2ns and 150ns respectively, obtained by extracting the slow component associated with a bi-exponential fit shown in Fig S2b.

In Fig. S3 we report the comparison between the time correlation of the total dipole and of the density within both the HDL and LDL phases of the SCAN-DFT model. While for HDL phase, density and polarization relax on similar timescales, in the LDL, the dynamics is significantly slower.

2. Filtering Time Series

To reduce the noise arising from high-frequency fluctuations in the dipole components, we applied a second-order low-pass digital butterworth filter (implemented using the SciPy.signal package in Python), engineered to maintain a near-flat frequency response within the passband. Sampling of the trajectories occurred every 5 picoseconds (ps), with a cutoff frequency set at 10GHz (1/100 in inverse ps), corresponding to the frequency at which the magnitude response of the filter reaches its threshold. In contrast to the room temperature, relaxation dynamics is much slower and hence one does not need a small time-step for processing the trajectories.

In addition to this initial filtering step aimed at reducing noise and preserving essential signal components, we further refined the trajectories by applying a mean filter using the *smooth* function with a span of 20 data points (equivalent to a time window of 100 ps). This additional smoothing procedure facilitated the subsequent automated detection of angular swings by ensuring smoother trajectories.

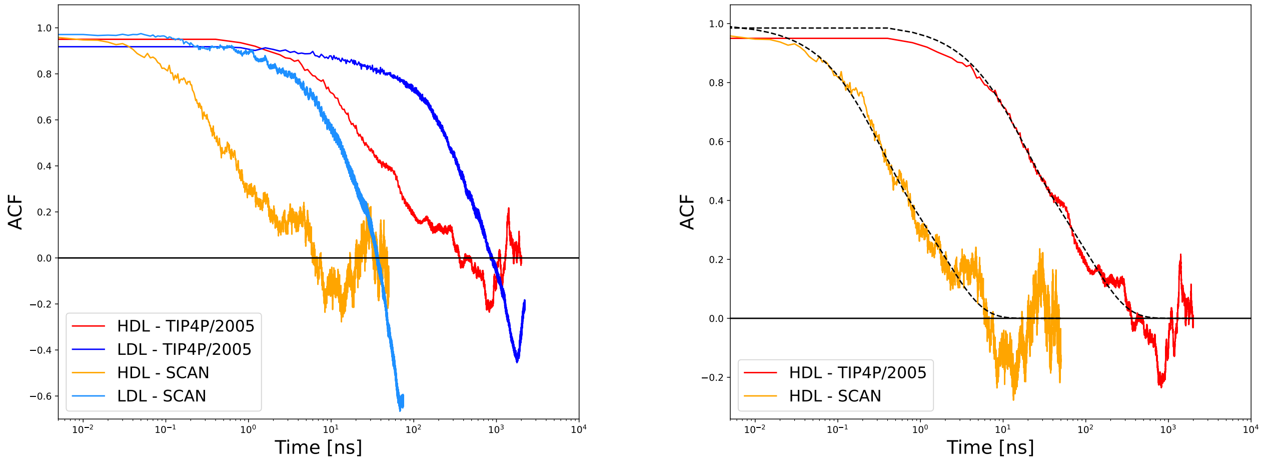

(a) (b)

Fig. S2. Left panel shows the total dipole correlation function computed for both TIP4P/2005 and SCAN-DFT models within low- and high-density supercooled water. Right panel shows the exponential fit to the dipole correlation of the HDL phase for the two models.

Moreover, during the post-processing phase of the defect fractions and the count of swings within the time series, we introduced an extra layer of filtering using a low-pass butterworth filter with a cutoff frequency of 0.05GHz (1/20000 in inverse ps). This supplementary filtering step enabled us to effectively capture fluctuations occurring on the nanosecond (ns) scale.

References

1. PG Debenedetti, F Sciortino, GH Zerze, Second critical point in two realistic models of water. Science **369**, 289–292 (2020).
2. TE Gartner, PM Piaggi, R Car, AZ Panagiotopoulos, PG Debenedetti, Liquid-liquid transition in water from first principles. Phys. Rev. Lett. **129** (2022).

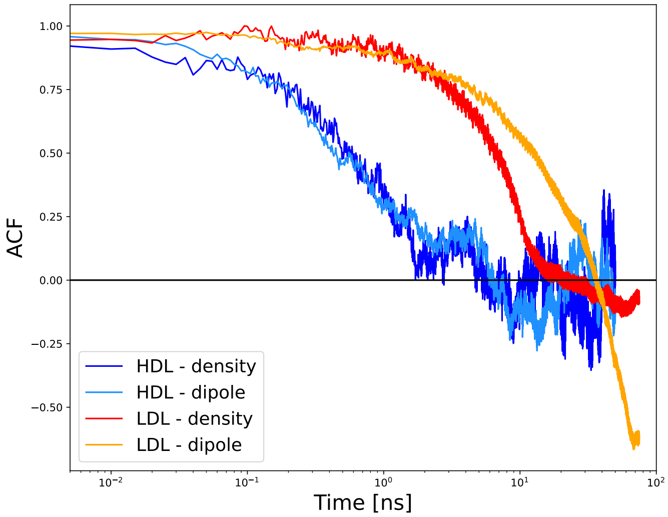

Fig. S3. Comparison between the total dipole time correlation function and the density time correlation function of the SCAN water model for both the HDL and LDL phases.

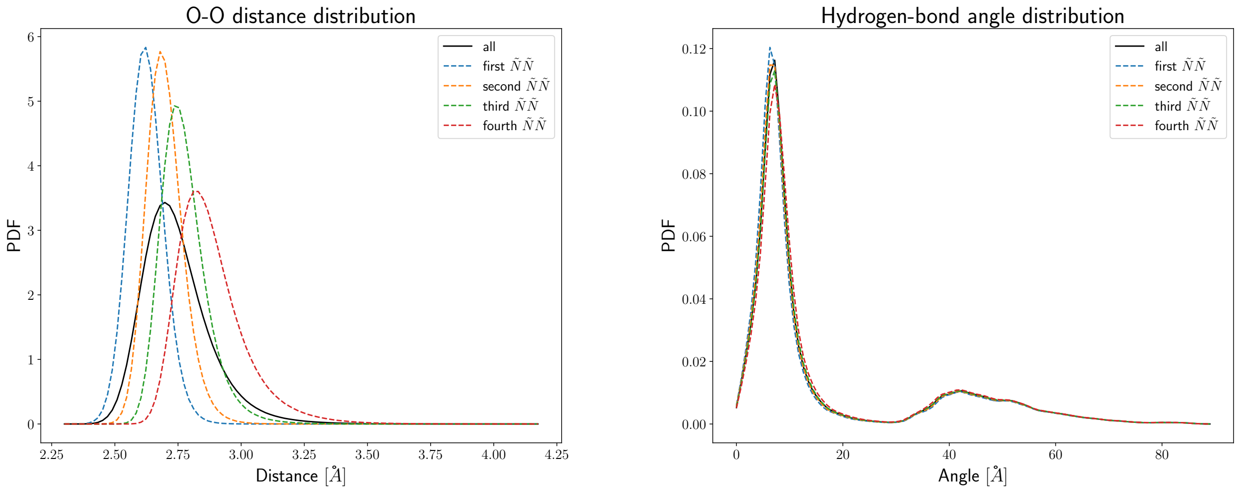

(a) (b)

Fig. S4. Left panel shows the oxygen-oxygen distance distribution function for the nearest 4 neighbours to every water molecule. Right panel shows the hydrogen-bond angle distribution for these 4 nearest neighbour waters.
